# *TP*53 and *MDM2 *gene polymorphisms and risk of hepatocellular carcinoma among Italian patients

**DOI:** 10.1186/1750-9378-6-13

**Published:** 2011-08-15

**Authors:** Valeria Di Vuolo, Luigi Buonaguro, Francesco Izzo, Simona Losito, Gerardo Botti, Franco M Buonaguro, Maria Lina Tornesello

**Affiliations:** 1 Molecular Biology and Viral Oncology and AIDS Ref. Centre, National Cancer Institute "Fond. Pascale", Naples, Italy; 2 Hepato-biliary Surgery Department, National Cancer Institute, "Fond. Pascale", Naples, Italy; 3 Department of Pathology, National Cancer Institute, "Fond. Pascale", Naples, Italy

**Keywords:** Genetic Polymorphisms, *TP*53 codon 72, *MDM2 *SNP309, hepatocellular carcinoma

## Abstract

**Background:**

Single-nucleotide polymorphisms within *TP*53 gene (codon 72 exon 4, rs1042522, encoding either arginine or proline) and *MDM2 *promoter (SNP309; rs2279744), have been independently associated with increased risk of several cancer types. Few studies have analysed the role of these polymorphisms in the development of hepatocellular carcinoma.

**Methods:**

Genotype distribution of *TP*53 codon 72 and *MDM2 *SNP309 in 61 viral hepatitis-related hepatocellular carcinoma cases and 122 blood samples (healthy controls) from Italian subjects were determined by PCR and restriction fragment length polymorphism (RFLP).

**Results:**

Frequencies of *TP*53 codon 72 alleles were not significantly different between cases and controls. A significant increase of *MDM2 *SNP309 G/G and T/G genotypes were observed among hepatocellular carcinoma cases (Odds Ratio, OR = 3.56, 95% Confidence Limits, 95% CI = 1.3-9.7; and OR = 2.82, 95% CI = 1.3-6.4, respectively).

**Conclusions:**

These results highlight a significant role of *MDM2 *SNP309 G allele as a susceptibility gene for the development of viral hepatitis-related hepatocellular carcinoma among Italian subjects.

## Introduction

Hepatocellular carcinoma (HCC) is the most common liver malignancy as well as the third and the fifth cause of cancer death in the world in men and women, respectively [1]. HCC is an infrequent cancer in developed countries, with the exception of Southern Europe where the incidence in men (ASR 9.8 per 100,000) is significantly higher than in other developed regions [2]. The HCC incidence, however, has substantially increased in Japan during the past three decades [3], and slight increases have been reported in France and in the United Kingdom [4,5]. The incidence rates of HCC in the United States have historically been lower than in many countries. However, in recent decades, HCC age-adjusted incidence rates have doubled [6].

Etiological factors associated with the development of the disease are well known and include infection with the hepatitis C virus or hepatitis B virus, heavy alcohol intake, prolonged dietary exposure to aflatoxin B1 and primary hemochromatosis [7-9]. As for other types of cancer, the etiology and pathogenesis of HCC is multifactorial and multistep [10]. The prevalence of HCC in Italy, and particularly in Southern Italy, is significantly higher compared with other Western countries. Hepatitis virus infection, long-term alcohol and tobacco consumption account for 87% of HCC cases in the Italian population and, among these, 61% are attributable to HCV infection. A recent seroprevalence survey conducted in the general population of Campania Region (Southern Italy) reported a 7.5% positivity for HCV infection which peaked at 23.2% in the 65 years or older age groups [11]. Thus, although the virus is highly prevalent only a small percentage of infected individuals will develop liver cancer suggesting that other genetic or environmental cofactors are needed for tumor progression.

Several studies have analyzed distribution of single nucleotide polymorphism in gene encoding for cell cycle regulatory proteins and susceptibility to malignant diseases. A common polymorphism located in exon 4 of *TP*53 gene, resulting in a non-conservative arginine to a proline change at codon 72, is known to be important for growth suppression and apoptotic functions [12,13]. Additionally, a naturally occurring G to T sequence variation (single nucleotide polymorphism - SNP309) in the second promoter-enhancer region of the *MDM2 *gene has been shown to increase the binding affinity of the transcriptional activator Sp1 resulting in high levels of *MDM2 *protein, formation of transcriptionally inactive p53-*MDM2 *complexes and alteration of the p53 pathway [14-16]. These observations are consistent with an oncogenic function for the variant SNP309 [17].

Polymorphisms of *TP*53 codon 72 and *MDM2 *SNP309 have been previously investigated in Korean population in which they are associated with the early development of HCC in patients with chronic HBV infection [18].

No studies have been conducted in HCC cases of the Italian population. In the present study, we evaluated the distribution of the *TP*53 codon 72 genotypes and *MDM2 *SNP309 in a series of 61 hepatocellular carcinoma cases comprising HCV and HBV infected patients, and compared them with healthy controls to possibly identify host genetic factors associated with the increased risk of developing hepatocellular carcinoma.

## Materials and methods

### Patient and Tissue Samples

Liver biopsies from 53 HCV-positive, 7 HBV-positive, one HCV/HBV-positive HCC patients and 122 negative non-liver cancer control patients (blood samples) were obtained with informed consent at the liver unit of the INT "Pascale" in Naples. Each liver biopsy was divided in two sections: the first section was stored in RNA Later at -80°C (Ambion, Austin, TX), the second was subjected to histopathologic examination. Only liver biopsies determined to be hepatocellular carcinoma were included in the study.

Genomic DNA was extracted according to published procedures [19]. Tissue samples were digested with by proteinase K treatment (150 μg ml-1 at 56°C for 2 hours) in 100 - 500 μl of lysis buffer (10 mM Tris-HCl, pH 7.6, 5 mM EDTA, 150 mM NaCl, 1% SDS), followed by DNA purification by phenol-chloroform-isoamyl alcohol (25:24:1) extraction and ethanol precipitation in 0.3 M sodium acetate (pH 4.6). Genomic DNA from 122 blood samples from healthy controls were previously extracted and subjected to allele specific PCR of exon 4 of *TP*53 and PCR-RFLP of intron 1 of *MDM2 *[20,21].

### *TP*53 codon 72 and *MDM2 *SNP309 polymorphisms analysis

The analysis of *TP53 *genotype at codon 72 was carried out by performing an allele specific PCR in which two independent reactions were performed for each sample: one specific for codon CCC (encoding proline) using the p53ProPlus (5'-GCCAGAGGCTGCTCCCCC-3') with p53Minus oligoprimers; the other specific for CGC (encoding arginine), using the p53Plus with p53ArgMinus (5'-CTGGTGCAGGGGCCACGC-3') oligoprimers. PCR amplification reactions were carried out on 100 to 200 ng genomic DNA in 50-μl reaction mixture following amplification procedures described previously [20].

The *MDM2 *SNP309 was determined by PCR and restriction fragment length polymorphism (RFLP) analysis of amplified products. A 174 base pairs (bp) amplimer of the *MDM2 *intron 1 region, containing the *Msp*A1 polymorphic site at nucleotide 309, was amplified with the B-MDM2-309F (5'- GGGAGTTCAGGGTAAAGG-3') and the B-MDM2-309R (5'- GACCAGCTCAAGAGGAAA-3') oligoprimers, designed using the Beacon Designer 2.0 software (Premier Biosoft International, Palo Alto, CA, USA). PCR reactions were performed in a 50-μl reaction mixture containing 100-200 ng of target DNA, 20 pmol of each primer, 2.5 mM MgCl_2_, 50 μM of each dNTP and 1.25 U of HotMaster *Taq *DNA polymerase (5 Prime GmbH, Hamburg, Germany). DNA was amplified with the following steps: an initial 1-min denaturation at 94°C, followed by 32 cycles of 55°C for 45 s, 68°C for 1 min, 94°C for 30 s and a final annealing at 55°C for 30 s with 5 min elongation at 72°C. Ten-microliter aliquots of *MDM2 *PCR products were digested with MspA1 restriction enzyme, fractionated by electrophoresis on a 7% polyacrylamide gel in Tris-borate-EDTA running buffer and stained with ethidium bromide for DNA band visualization by UV transillumination.

*TP*53 PCR amplified products from the 61 cases were further subjected to direct nucleotide sequencing by Primm Srl Laboratories (Milan, Italy) using the fluorescent dye terminator technology and ABI 3730 DNA sequencers (Applied BioSystems). Nucleotide sequences were edited with Chromas Lite 2.01 (http://www.technelysium.com.au/chromas.html).

### Statistical analyses

The observed and expected genotype frequencies among the study groups were analysed using the Hardy-Weinberg equilibrium theory. A Fisher's exact test or χ^2 ^test was used, as appropriate, to compare the proportions of *TP*53 and *MDM2 *genotypes between cases of HCC and healthy control population. An unpaired Student's *t*-test was used to evaluate differences between the mean age of cases and control group. All analyses were performed with Epi Info 6 Statistical Analysis System Software (6.04d, 2001, Centers for Disease Control and Prevention, USA).

Differences were considered to be statistically significant when *p-*values were less than 0.05.

## Results

In this study were included 61 cases of hepatocellular carcinoma (48 in males and 13 in females) and 122 cancer-free control subjects (all males). Table 1 summarizes the selected characteristics of the subjects. The frequencies of *TP53 *codon 72 genotypes has been estimated using an allele specific PCR that specifically detects either the *TP*53 proline or arginine 72- encoding codons. A slight higher frequency of arginine allele was observed in HCC cases compared to the controls, however, the difference did not reach statistical significance using Yates corrected *χ*^2 ^test (*P *= 0.696). The distribution of polymorphic alleles in cases and controls are summarized in Table 2. The frequencies of the three genotypes of the *TP53 *gene polymorphism at codon 72 among the 61 HCC cases were 4.9% (n = 3), 32.8% (n = 20) and 62.3% (n = 38) proline homozygous, heterozygous and arginine homozygous, respectively, and the corresponding figures among controls were 7.4% (n = 9), 34.4% (n = 42), and 58.2% (n = 71), respectively. The genotype frequencies of *TP*53 codon 72 of were found in Hardy-Weinberg equilibrium both in HCC cases and healthy controls (*χ*^2 ^*= *0.81; df = 1; *p *= 0.37; and *χ*^2^*= *0.63; df = 1; *p *= 0.43, respectively).

**Table 1:** Characteristics and pathological features of HCC patients and healthy controls

Variable	Cases (n = 61) n(%)	Controls (n = 122) n(%)
		
Mean age, years [± SD]	68.7[± 8.87]	59.4[± 14.3]
		
Age, years		
≤ 50	2(3.3)	12(13.1)
50-65	16(26.2)	43(45.9)
> 65	43(70.5)	38(41)
		
Males	48(78.7)	122(100)
Females	13(21.3)	
		
Virus		
Cases HCV-pos	53(86.9)	
Cases HBV-pos	7(11.5)	
Cases HCV/HBV-pos	1(1.6)	

**Table 2:** Distribution of *TP*53 codon 72 and *MDM*2 SNP309 genotypes in HCC cases and controls

	HCC Cases (n = 61) n (%)	Controls (n = 122) n (%)	OR^a ^(95% CI)	*P *Value^b^
***TP*53 codon 72**				
*Pro *Allele	27(22.1)	60(24.6)	1	
*Arg *Allele	95(77.9)	184(75.4)	1.15(0.7-2.0)	0.695
				
*Pro/Pro*	3(4.9)	9(7.4)	1	
*Pro/Arg*	20(32.8)	42(34.4)	0.96(0.2-4.3)	0.875
*Arg/Arg*	38(62.3)	71(58.2)	1.20(0.3-5.0)	0.716
*Arg/Arg + Pro/Arg*	58(95.1)	113(92.6)	1.12(0.3-4.5)	0.751
				
***MDM2 *SNP309**				
T Allele	58(47.5)	154(63.1)	1	
G Allele	64(52.5)	90(36.9)	1.89(1.2-3.0)	0.006
				
T/T	13(21.3)	55(45.1)	1	
T/G	32(52.5)	48(39.3)	2.82(1.3-6.4)	0.010
G/G	16(26.2)	19(15.6)	3.56(1.3-9.7)	0.009
G/G + T/G	48(78.7)	67(54.9)	3.03(1.4-6.6)	0.003

^a^T allele or T/T *MDM2 *SNP309 and *TP*53 *Pro *allele or *Pro/Pro *codon 72 genotypes were considered as the baseline when calculating the relative crude ORs.

^b^Yates corrected P values

**Figure 1:**
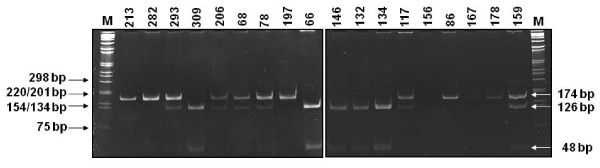
**PCR amplification followed by restriction fragment length polymorphism (PCR-RFLP) to determine *MDM*2 SNP309 polymorphism**. *MDM*2 SNP309 T allele was not cleaved by *Msp*AI endonuclease and had a single band of 174 bp. The *MDM*2 SNP309 G allele was cleaved by *Msp*AI and had two small fragments of 126 and 48 bp. The *MDM*2 SNP309 heterozygote had three bands of 174, 126 and 48 bp.

The *MDM2 *SNP309 genotypes were analyzed using a PCR-RFLP based assay (Figure 1). The frequencies of *MDM2 *SNP309 T/T, T/G and G/G genotypes among the cases were 21.3% (*n *= 13), 52.5% (*n *= 32) and 26.2% (*n *= 16), and among controls were 45.1% (*n *= 55), 39.3% (*n *= 48) and 15.6% (*n *= 19), respectively. The *MDM2 *SNP309 genotype frequencies were found in Hardy-Weinberg equilibrium among cases and controls group (*χ*^2 ^*= *0.16; df = 1; *p *= 0.69; and *χ*^2^*= *2.33; df = 1; *p *= 0.13, respectively). The frequencies of *MDM2 *SNP309 G/G and G/T genotypes were significantly more prevalent in HCC cases (52.5%) compared with controls (36.9%), *p *= 0.006. When the homozygous *MDM2 *SNP309 T/T genotype was used as the reference group, the *MDM2 *SNP309 G/G and T/G genotypes were associated with a significantly increased risk for HCC (OR 3.56, 95% CI 1.3-9.7; OR 2.82, 95% CI 1.3-6.4, respectively).

The combined effect of the TP53 codon72 and the MDM2 SNP309 polymorphisms was also analysed. However, no additional cancer risk effect associated with TP53 codon 72 polymorphism was identified when the risk of combined genotypes was analyzed.

## Discussion

Several epidemiological studies have evaluated the association of *TP*53 codon 72 and MDM2 SNP309 polymorphisms and risk of or survival from several types of cancer [22-24], but few evaluated the association of both these polymorphisms in viral-related liver cancer and none in hepatocellular carcinoma from Italian patients.

In the current study the *TP*53 codon 72 and *MDM2 *nucleotide 309 polymorphisms were investigated in a series of 61 cases of hepatocellular carcinoma, mainly associated with HCV infection, and in 122 population-matched controls in order to verify the impact of these gene variants on the risk of tumour development. The *TP*53 codon 72 and MDM2 SNP309 genotypes were distributed in accordance with the Hardy-Weinberg equilibrium in controls and their frequencies were in broad agreement with those observed in previously published studies of Caucasian subjects [25-27]. The results showed no significant differences in the genotype distribution of the *TP*53 codon 72 polymorphisms between cases and controls and no increase in the frequency of the arginine or proline alleles among HCC cases. This observation is in agreement with previous studies reporting no association between codon 72 genotypes and disease severity or liver cancer in a group of 340 HCV-infected Italian subjects at different stages of disease, including 84 HCC patients, and in a group of 97 Spanish HCC patients [28,29]. Conversely, a higher frequency of Pro/Pro genotype in HCC cases (13.5%) versus control subjects (6.3%) and a significant higher risk to develop liver cancer (OR, 2.304; 95% CI, 1.014-5.234) was observed among Moroccan subjects [30]. The analysis of pooled data from six case-control studies including 836 and 279 liver cancer and 1'191 and 587 controls of Asian and Caucasian ethnicity, respectively, showed a significant higher risk (OR = 1.35, 95% CI 1.06 - 1.71) for liver cancer only among Asian patients carrying the *Pro/Pro *genotype [31]. A case-control study including 80 incident cases of HCC and 328 controls, nested in a cohort of 4,841 male chronic hepatitis B, showed that the lack of association between *TP*53 codon 72 *Pro *allele and HCC was apparent. Indeed, in patients with chronic liver disease there were synergistic effects between the *Pro *allele and HCC family history in first-degree relatives which conferred a risk of 7.60 (95% CI = 2.28-25.31) for HCC development compared with subjects without the *Pro *allele and without chronic liver disease [32]. These observations suggest that large case-control studies are needed in order to clarify the role of *TP*53 codon 72 polymorphism in the HCC pathogenesis in different populations.

In this study the frequencies of *MDM2 *SNP309 T/G heterozygous (52.5%) and G/G homozygous (26.2%) genotypes were significantly higher among HCC cases compared to healthy controls (P 0.010 and P = 0.009, respectively). The homozygous MDM2 SNP309 T/T genotype in HCC, on the other hand, was lower (21.3%) than that observed in controls (45.1%). The possible role of *MDM2 *SNP309 polymorphism in HCC development has been analyzed in few other geographical regions. In Eastern Asia *MDM*2 SNP309 G allele has been associated with higher risk of HCC in Japanese patients with chronic hepatitis C [33], in Korean patients with chronic HBV [18], and in Taiwanese patients infected with HBV or HCV infection [34]. Similarly, the MDM2 309 G allele was found to enhance the risk of viral-associated HCC in Moroccan (Northern Africa) [35] and in Turkish (Western Asia) patients [36]. Jin *et al. *(2011) performed a metanalysis on these studies, including a total of 738 cases and 1062 controls, to evaluate the reliability of the associations of *MDM2 *SNP309 G allele with HCC by using false-positive report probability analysis and the Venice guidelines on genetic epidemiology [37]. The pooled OR reached 1.57 (95% CI: 1.36-1.80) for G allele compared to T allele, using the fixed-effect model, indicating that this SNP may be used as a predictive molecular marker for the screening of populations with high risk of HCC [37].

Overexpression of the MDM2 protein has an oncogenic effect through the reduction of p53 levels with the consequent attenuation of the p53 DNA damage response that allows increased cell proliferation and inhibition of apoptosis, providing advantageous signals for tumor cell survival [38-40]. The study by Bond et al. (2004) showed that MDM2 protein levels in homozygous and heterozygous SNP309 G cell lines are on average 4-fold and 1.9 fold higher, respectively, than TT cell lines [14]. Furthermore, Hirata et al. reported that renal cell carcinoma tissue with G/G and G/T genotypes were more frequently positively stained for MDM2 than that with the TT genotype (50% and 26%, respectively, versus 13%) [41]. The proper regulation of MDM2 levels has been shown to be critical for p53 tumour suppression, and even a modest change in levels could affect the p53 pathway and, subsequently, increase the risk of cancer development in mouse models [42]. MDM2 binds directly to and inhibits p53 by regulating its location, stability and ability to activate transcription [14]. Several reports have shown that HCV proteins (NS3, NS5A and NS5B) can modulate activity and/or expression of p53 and may interfere with normal regulation of cell growth [43-45]. This implies that MDM2 polymorphism together with viral hepatitis protein may result in a cumulative effect on the inactivation of p53 function.

This study has limitations, including the modest sample size and the fact that cases were not well matched for age and sex with the control subjects. Although Italian blood donors may be good representatives of the general population, they are younger than HCC patients due to age limit and health requirements for blood donation. However, being fulfilled the Hardy Weinberg equilibrium for the allelic frequencies of both *TP*53 and MDM2 polymorphisms in the control group, it may be excluded that age and sex limitations have introduced significant bias in the study.

In conclusion, these results provide the first evidence that the MDM2 SNP309 polymorphism represents a risk factor for viral hepatitis-related HCC in the Italian population. Conversely, the *TP*53 polymorphism at codon 72 seems not to be a potential risk factor for development of HCC in Italian patients.

## Abbreviations

HCC: Hepatocellular carcinoma; *MDM2*: mouse double minute-2; HBV: hepatitis B virus; HCV: hepatitis C virus.

## Competing interests

The authors declare that they have no competing interests.

## Authors' contributions

MLT and FMB were responsible for the overall planning and coordination of the study. FI was involved in patients enrollment and specimen collection. LB contributed to the data analysis. SL and GB carried out the histopathology evaluation of the cases. VDV was responsible for specimen processing, DNA analysis and with MLT compiled and finalized the manuscript. All authors read and approved the final manuscript.
